# Investigation on Tattoo Ink (Hexadecachlorinate Copper Phthalocyanine) Removal: Novel Chemical and Biological Approach

**DOI:** 10.3390/molecules29235543

**Published:** 2024-11-24

**Authors:** Giancarlo Ranalli, Alessia Andreotti, Maria Perla Colombini, Cristina Corti, Debora Paris, Laura Rampazzi, Gabriella Saviano, Ramona Vecchio, Claudio Caprari

**Affiliations:** 1 Department of Biosciences and Territory, University of Molise, c.da Fonte Lappone snc, 86090 Pesche, Italy; ranalli@unimol.it (G.R.); saviano@unimol.it (G.S.); ramona.vecchio1@gmail.com (R.V.); 2 Department of Chemistry and Industrial Chemistry, University of Pisa, via Giuseppe Moruzzi 13, 56124 Pisa, Italy; alessia.andreotti@unipi.it (A.A.); maria.perla.colombini@unipi.it (M.P.C.); 3 Department of Human Sciences, Innovation and Territory, University of Insubria, via Sant’Abbondio 12, 22100 Como, Italy; cristina.corti@uninsubria.it (C.C.); laura.rampazzi@uninsubria.it (L.R.); 4 The Institute of Biomolecular Chemistry, National Research Council of Italy, via Campi Flegrei 34, 80078 Pozzuoli, Italy; dparis@icb.cnr.it; 5 The Institute of Heritage Science, National Research Council of Italy, via Roberto Cozzi 53, 20125 Milan, Italy

**Keywords:** tattoo ink removal, Py-GC/MS, NMR, FTIR, *Pseudomonas stutzeri*, *Alternaria infectoria*

## Abstract

Tattoos have been a ubiquitous phenomenon throughout history. Now, the demand for tattoo removal for aesthetic or practical reasons is growing rapidly. This study outlines the results of field investigations into the chemical and biological removal of tattoo inks (Hexadecachlorinate copper phthalocyanine—C_32_Cl_16_CuN_8_—CAS no° 1328-53-6). FTIR, Py-GC/MS, and NMR analyses yielded intriguing profiles pertaining to the primary chemical constituents, along with others of an ambiguous nature. A bioremoval protocol was developed on a pork rind surface to simulate human tattooing. Two previously studied microbial strains were included in this analysis: (i) a bacterial culture of *Pseudomonas stutzeri* 5190 DSMZ viable cells and (ii) a fungal culture of *Alternaria infectoria* strain NIS4, the latter already isolated and identified. A combination of physical, chemical, and microbiological analyses, along with microscopic observations, was conducted. In our experimental conditions, inocula from environmental samples (soil and compost) were capable of inducing changes in even trace organic matter (glycerin and additives in pigments) used as a binder in emulsifiers in tattoo inks. Furthermore, the two microbial strains demonstrated promising potential for removing green tattoo ink. Finally, wastewater effluents containing green ink were recovered via electrochemical treatment, and the environmental impact in terms of the CO_2_ equivalent of our experiments was assessed. The results are promising and warrant further investigation into the innovative biological and chemical removal of tattoo inks from human skin and wastewater, respectively.

## 1. Introduction

Tattoos have been a ubiquitous phenomenon throughout history, serving a multitude of religious, social, and aesthetic purposes [1,2]. A review of historical sources reveals a long history of tattooing practices; see Table 1 [1].

In recent decades, the prevalence of tattoos has increased significantly among the young and total human populations [3,4,5,6], reaching values up to 24% in the USA [4] and less than half that in Europe, with approximately 9% in Germany and 12% in the UK and Italy [7,8,9].

**Table 1:** Ancient and traditional tattoo practices.

When	Where (Country)	Who	Why (Significance)	Refs
Ancient and traditional use	Greece, Rome, Italy, and Austria (Alps)	Otzi	Mummified human	[10]
	North America	Indigenous People	Skin modifications	[11]
		Inuit	Symbolize a girl transitioning into a woman	[12,13]
		Osage People	Man, as smaller cycle of life	[14]
		Iroquois People	Cultural significance of the Manitou—Warfare	[15]
	Central America			
	Taiwan			
	Philippines		Head hunting	[16,17]
	Solomon Islands		Historical events	[17]
	Japan	Samurai, Criminals	Samurai’s bravery and loyalty; offenses of the underworld	[18]
	China		Various aspects of social life	[19]
	Egypt Nubia, Copts		Religion aspects	[20]
Modern tattooing	Reintroduction to the Western world pre-1860s	British Court British Sailors		[21]
	Early United States		The Tattoo Renaissance	[22]
		Indigenous Boarding School Tattooing Practices		[23]
Military sectors	United States		Luck, protection, wartime experience, or enhance their combat abilities	[24,25]

Among the various chemical compounds utilized in tattoo inks, azo pigments are particularly prevalent due to their stability over time and color intensity.

The chemical composition of tattoo inks is based on mixtures of colored pigments and a variety of additional components. These pigments may contain diluents, including both polar and non-polar solvents, which are necessary for pigment dilution and useful for the final application of the surface [26,27,28].

Tattooing involves transferring and placing inks and pigments in the dermis using tiny rigid needles. Given the unique nature of the process, each tattoo carries an inherent risk to the skin owing to the introduction of foreign substances. Adverse skin reactions, including those affecting the tattooed surface and other anatomical locations such as lymph nodes, have been observed in some cases owing to the presence of pigments, impurities, and other factors [6,29,30,31,32,33,34,35,36,37,38]. Furthermore, carcinogenic amines can be formed as a result of prolonged exposure to ultraviolet radiation from solar light [39,40]. European legislation has taken a more cautious approach than other countries in recognizing the potential risks associated with certain pigments. This has led to their prohibition in cosmetics, including Pigment Red 22, owing to the proven composition of their constituent ingredients [27].

The colors utilized in early tattooing were likely derived from natural sources, such as carbon-based black, plant, and animal organic pigments. Over the past century, synthetic organic and inorganic pigments have emerged as a prominent alternative, offering a vast array of hues. These pigments frequently comprise azo compounds, oxazines, phthalocyanines, and mineral oxides [2]. Regardless, all pigments contain chromophores, such as azo, carbonyl, and thiol functional groups.

Tattoos remain a popular form of body art, and the demand for tattoo removal for aesthetic or practical reasons necessitates characterizing the materials used prior to the procedure. This is achieved through mechanical abrasion with salts, sandpapers, brushes, fraises, and laser treatment, which is highly effective, as it selectively removes chromophores [41]. The ease of removal is contingent upon the ink utilized. For instance, the literature indicates that green tattoo inks are among the most challenging to remove via laser because of their chemical composition [42]. In addition to articles that assess the medical harmfulness of tattoo inks, the primary focus of tattoo literature is to identify the ink, which may be of forensic interest in cases of unidentified human remains. In this context, data pertaining to the age, temporality of removal, and provenance of ink residues at the crime scene are of significant utility [43].

**Figure 1:**
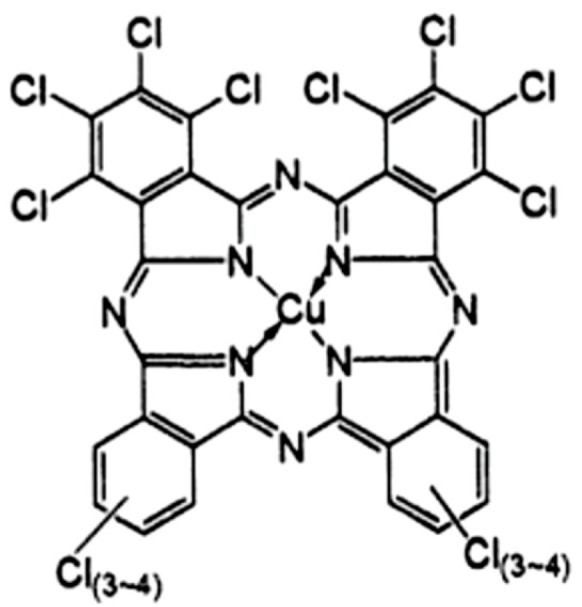
Chemical structure of Cyanine Green G related to the green ink tattoo in this study [46].

Copper phthalocyanine green (CAS n° 1328-53-6) is a synthetic green pigment belonging to the phthalocyanine dye family (Figure 1). Chlorine atoms affect the electron distribution within phthalocyanine molecules. Chlorine atoms in copper phthalocyanine blue shift the ink’s color to green [44]. Phthalo green is alkali-, acid-, solvent-, heat-, and UV-radiation-stable. It is suitable for inks, oils, coatings, and plastics [45]. Green tattoo ink (Hexadecachlorinated copper phthalocyanine) is a compound, which, due to its peculiar chemical structure including a central Cu ion with 16 Cl molecules, shows great environmental stability (water, soil, and wastewater) with very low degradation. Only a few degradation methods, such as the Fenton reaction (oxidation of organic pollutants by hydrogen peroxide and iron (II), ozone reagent), are useful when applied directly to wastewater and residues containing similar xenobiotics.

The composition of these inks is unknown, as manufacturers do not provide accurate compositions of the materials on labels or data sheets. In their review, Miranda et al. reported the most commonly used analytical techniques for determining pigments [2].

The biological method tested and proposed acts on the surface of animal tissue used as a laboratory model (pork rind). In this case, the metabolic action of a pool of enzymes in mixtures activated by two selected microorganisms (*P. stutzeri* and *A. infectoria*, added separately) promotes the removal of the ink.

Once our experiment is complete, our team will assess the environmental impact of the methodologies used to propose solutions to help recover the xenobiotic-polluting compounds used and mitigate their environmental effects.

The objectives of this study are to (i) perform a chemical analysis of the inks commonly used in tattooing; (ii) investigate the potential use of microorganisms as a novel and alternative method for tattoo removal; (iii) evaluate the efficacy of an electrochemical treatment of wastewater inks; and (iv) assess the environmental impact in terms of the CO_2_ equivalent of our experiments to further mitigate actions.

## 2. Results and Discussion

### 2.1. FTIR-ATR Analysis of Inks

FTIR analysis focused on the pigments contained in the inks and the qualitative and quantitative changes to assess the effects of the treatments.

The black ink “Makkuro Sumi Pure Black” contains a single pigment, carbon black, whose spectrum may exhibit signals attributable to organic substances. Since it is impossible to uniquely impute these signals to the pigment or organic additives present in the ink, IR spectra were not considered reliable for monitoring any changes in the ink.

The same does not apply to the green ink “Azayaka True Green dispersion”, which contains Pigment Green 7 (PG7) and Pigment Yellow 1 (PY1). PG7 is composed of polychlorinated copper phthalocyanine, while PY1 comprises the dye “Hansa Yellow G”, which belongs to the monoazo dye class.

**Figure 2:**
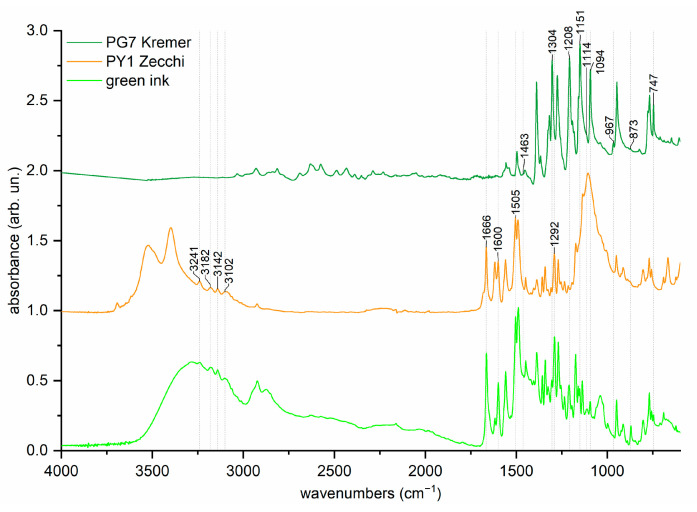
FTIR-ATR spectra of green ink “Azayaka True Green dispersion”, PG7 “Kremer 23000 Phthalo Green dark” and PY1 “Zecchi Primary Hansa Yellow”. Diagnostic peaks are reported on the spectra.

The FTIR-ATR spectrum of the ink is shown in Figure 2 compared with those of the PG7 Kremer “23000 Phthalo Green Dark” and PY1 “Zecchi Primary Hansa Yellow” pigments as references. The spectra were normalized to the peak with the highest intensity to make differences more evident.

The typical peaks of the PG7 spectrum can be found in the literature, with some peaks assigned, a few of which are diagnostic for its identification. In particular, peaks at 1389, 1304, 1207, 1159, 1094, and 949 cm^−1^ (all attributable to C-H in-plane vibrations), 768 cm^−1^ (C-Cl stretching vibrations), and 1497, 1454, 1317, 1192, 1182, 1149, 968, 648, 631, 606, and 509 cm^−1^ are identified as relevant in the literature [41,47,48,49]. Table S1 in the Supplementary Material reports the actual experimental values of peaks that can be attributed to one of the reference pigments, either PG7 or PY1.

The spectrum of the reference pigment PY1 “Zecchi Primary Hansa Yellow” shows intense gypsum peaks (3522, 3399, 1679, 1618, 1107, 1136, 1005, and 667 cm^−1^), as well as less intense peaks that could be attributed to kaolinite (3694, 3647, 3619, 1023, and 914 cm^−1^) and calcite (1405, 877, 712 cm^−1^) [50,51]. These components may have been added to the commercial pigment, which was prepared for use in art, to provide consistency and adjust the color. Figure 2 compares the spectrum with that of green tattoo ink.

Some of the peaks of PY1 are assigned in the literature, indicating which are diagnostic. Peaks at 3243, 3181, 3145, 3098, 1667 (attributed to carbonyl group vibrations), 1602, 1562, 1508, 1296, 953, and 774 cm^−1^ have been identified as relevant [52,53,54,55,56]

### 2.2. Microorganisms and Enrichment Culture Technique

#### 2.2.1. Enrichment Test Culture

**Figure 3:**
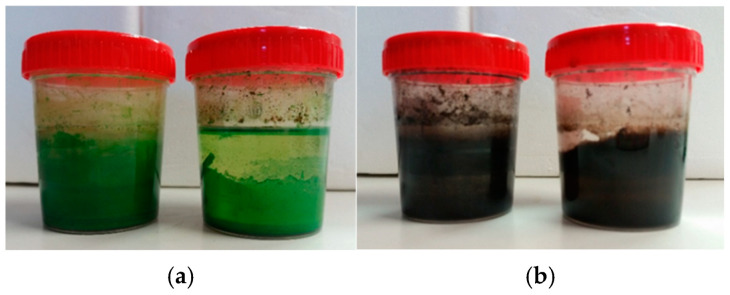
Example of enrichment test cultures performed on green (**a**) and black (**b**) ink tattoos.

Enrichment culture is an isolation technique designed to create very favorable growth conditions for an organism of interest while at the same time creating an unfavorable environment for any competitors. This is usually achieved by introducing nutrients or environmental conditions that allow only the desired organism to grow [57]. In our experiments, two sets of trials were performed. In the first, a strain of *Pseudomonas stutzeri* DSMZ 5190 [58,59] and a strain of *Alternaria infectoria* NIS4 [60] were used. In addition, two environmental samples, a soil sample (S) and a composted sample (C), were used as microbial inocula for enrichment culture techniques (Figure 3). Two grams of soil were added to black and green ink resuspensions (1 mL of ink suspension in 50 mL of mineral medium M9). The analyses performed on the “Azayaka True Green Dispersion” ink used for enriched cultures are described below.

#### 2.2.2. Microbial Community Analysis

After green and black inks were added over 12 months, the 16S rRNA gene amplicons (V3-V4 regions) of the soil and composted residues enriched with bacterial and fungal communities were subjected to NGS (Next-Generation Sequencing) analysis.

The results were unsatisfactory, as the requisite amounts of DNA for community investigation and study could not be recovered. The presence of complex, toxic substances may be a contributing factor, as they can inhibit the regular purification and amplification processes. The sequencing process and the resulting yield were inadequate. Despite taking purification steps with the samples, whether in their original state or diluted, the information was not timely.

#### 2.2.3. Py-GC/MS Analysis on “Azayaka True Green Dispersion”

The green ink sample showed several pyrolytic fragmentation peaks typical of phthalocyanine, including many aromatic and heterocyclic compounds [43]. Glycerol was also present, while natural gum was absent. A comparison with sample S (soil) shows that most of the characteristic markers disappeared or decreased, and glycerol disappeared.

**Figure 4:**
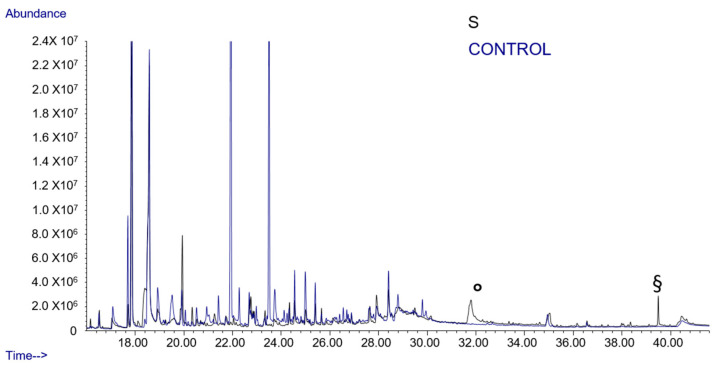
Pyrogram of sample S (soil) compared with control peaks (raw green ink).

Figure 4 reports pyrograms of sample S (soil) and the green ink sample (control), showing good similarity except for two peaks: one benzene-substituted imidazole (°) and a long-chain alkene (§).

**Figure 5:**
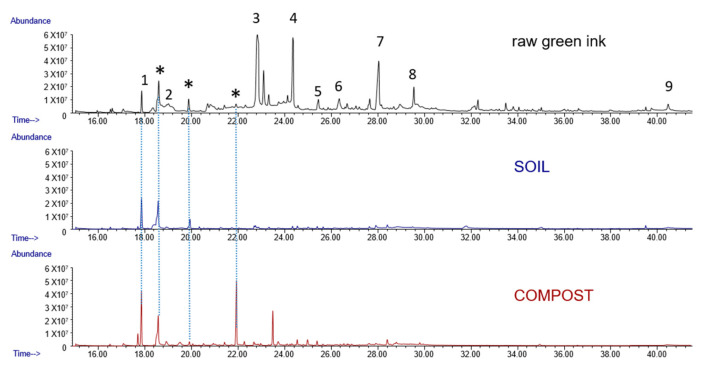
Pyrogram of sample S (SOIL) and C (COMPOST) compared with control peaks (green ink). Label with asterisk (*) belongs to byproducts of the derivatizing agent (HMDS). 1: chloro pentylphenol; 2: aniline; 3: glycerol (2TMS); 4: glycerol (3TMS); 5: quinolinamine; 6: phenylacetamide; 7,8: methyl nitro-benzenamines; 9: benzonitrile.

Pyrograms of the raw green ink sample (control) compared with sample S (soil) and sample C (compost) are reported in Figure 5. The main pyrolysis products of this green phthalocyanine have been identified, including different chlorinated heterocyclic and cyano-nitro compounds [61].

#### 2.2.4. NMR Analysis

**Figure 6:**
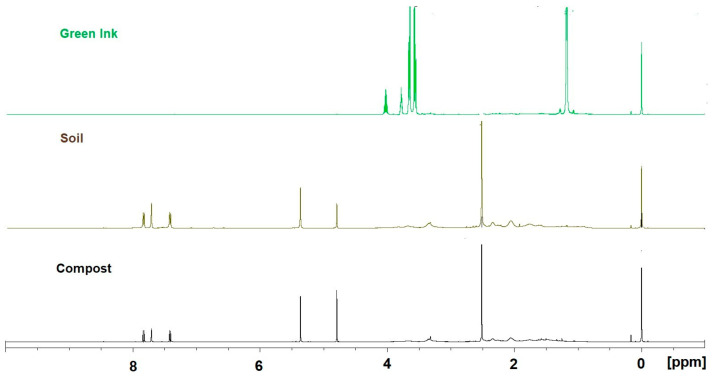
Comparison between 1D NMR experiments of raw green ink (**top**), soil after treatment with green ink (**middle**), and compost after treatment with green ink (**bottom**).

Figure 6 shows the 1D proton NMR spectra of aqueous extracts from the compost and soil after the ink treatment and raw green ink in D_2_O. In the raw green ink spectrum (top), only glycerol (3.56 *dd*, 3.65 *dd*, and 3.78 *m* ppm) and isopropanol (1.18 *d* and 4.02 *sep* ppm) signals were relevant; PG7 does not contain hydrogens (C_32_Cl_16_CuN_8_), and therefore, 1D NMR does not detect it. In addition, in the aliphatic region (3 ÷ 1 ppm), impurities are evident because of poorly soluble components. Instead, the 1D spectra of the compost and soil after the ink treatment appeared to be very different. Glycerol and propanol disappeared, and other components became evident. In particular, the chemical shifts at 7.41 (*d*), 7.71 (*s*), and 7.83 (*d*) ppm can be correlated with a substituted imidazole (or to any related compound). The correlation between the chemical shifts was supported by 2D TOCSY experiments (Figure S1). Also evident in these spectra are poorly soluble residues that cannot be identified in the aliphatic region of the spectrum. In addition, a peak at 5.36 ppm (*s*) could be attributed to methylene chloride. This statement is supported by the 1D spectrum of a minimal amount of methylene chloride dissolved in D*_2_*O.

**Figure 7:**
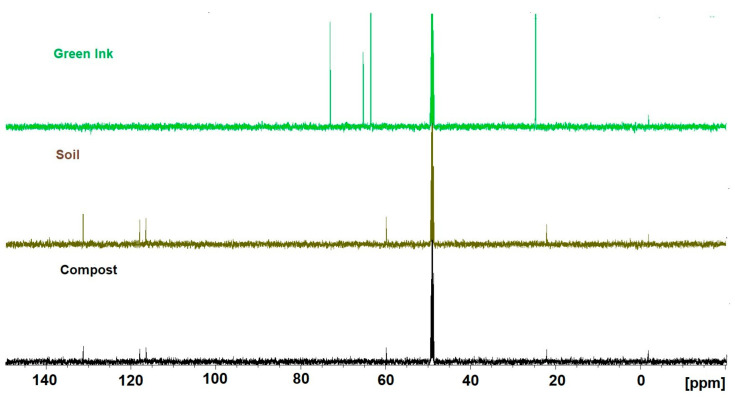
Comparison between 1D-^13^C NMR experiments of raw green ink (**top**), soil after treatment with green ink (**middle**), and compost after treatment with green ink (**bottom**).

^13^C NMR experiments confirmed the indications obtained from the 1H spectra. Figure 7 shows the 1D spectra of all three compounds.

The green ink ^13^C spectrum confirms the presence of glycerol, 63.5 ppm (C_1,3_) and 73.1 ppm (C_2_), and isopropanol, 24.8 ppm (C_1,3_) and 65.3 ppm (C_2_); the presence of the diamagnetic metal Cu at the center of the phthalocyanine affects the relaxation time of ^13^C and, thus, the experiment’s acquisition time. It also influences the normal chemical shift. For this reason, peaks related to the green ink compound are not evident. The soil ^13^C experiment and the compost ^13^C experiment were very similar. The chemical shifts at 116.4 ppm, 118 ppm, 131 ppm, and 22 ppm can be related to the presence of a substituted imidazole (or to any related compound). The c.s. at 59.9 is related to a proton identified as dichloromethane through an HSQC experiment (Figure S2).

#### 2.2.5. FTIR-ATR Analysis on “Azayaka True Green Dispersion”

**Figure 8:**
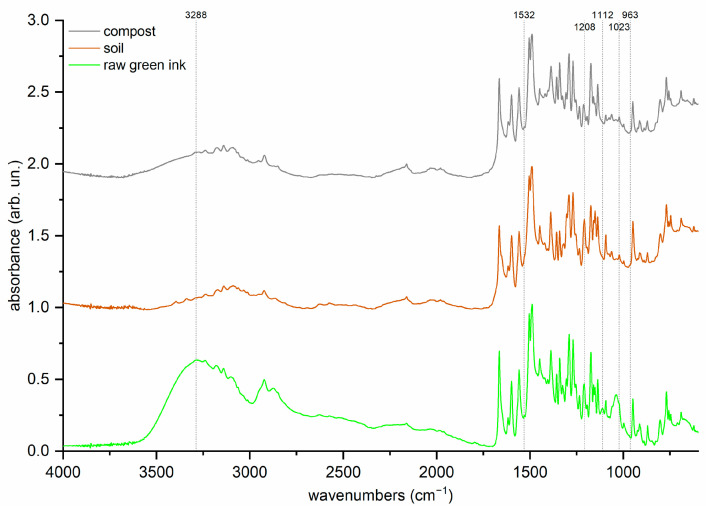
FTIR-ATR spectra on raw green ink (control), compared on soil and compost samples after separate addition of inocula.

The “Azayaka True Green Dispersion Ink” samples were analyzed before and after treatment. Figure 8 shows the green ink spectra before and after being added to the compost and soil samples, normalized to the peak with the highest intensity to make differences more evident.

In general, decreased intensity in all ink signals was observed after both treatments, but some bands disappeared. The spectral range that changed most was above 2000 cm^−1^. The most notable change seems to be the almost complete disappearance of the broad peaks at 3288 and 1023 cm^−1^, attributable to the ink additive, probably glycerol. In the same range, the signals of the PG7 and PY1 pigments (2924, 2874, and 2850 cm^−1^) significantly decreased in intensity, mostly attributable to C-H vibrations.

As for the rest of the spectrum, some peaks disappeared or mostly disappeared depending on the treatment. After both treatments, the signals at 1112 cm^−1^ (in-plane CH bending) and 963 cm^−1^ (out-of-plane CH bending) attributable to the PG7 pigment disappeared [41,46,62,63]. The post-soil-inoculum spectra show the loss of the 1532 cm^−1^ peak (aromatic CN stretching and CNC bending) of the PY1 pigment [48,49,52,53,54]. Conversely, the pigment PG7 signal at 1208 cm^−1^ (in-plane CH vibrations) disappeared after the compost inoculum [41,46,62,63].

### 2.3. Biological Removal of Inks

The microbial culture enrichment technique was employed to isolate microorganisms from environmental samples with specific functionalities, including the production of degrading enzymes and the ability to detoxify complex molecules [64]. After 12 months of enrichment, a total viable aerobic microbial count was performed on Potato Dextrose Agar (PDA) media to assess any bacteria and fungi. Bacterial and fungal cultures could not be isolated from the soil and compost samples. No growth was observed. The viable microbial count was 0 CFU/mL (Figure S3). These results were surprising given that numerous liquid enrichment tests revealed changes in the M9 growth substrate, which could be attributed to the interaction between microbial viable forms and the presence of inks. It is important to consider the possibility of viable but non-culturable microorganisms on standard growth substrates.

**Figure 9:**
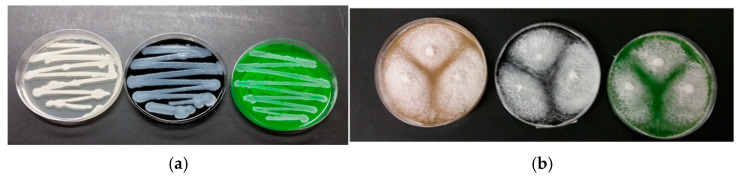
Microbial growths of *P. stutzeri* 5190 (**a**) and *A. infectoria* NIS4 (**b**) on PCA and PDA media with the addition of tattoo carbon black and green inks, respectively.

Table 2 and Figure 9 summarize the growth of pure and enriched microbial cultures.

Figure 9 illustrates the microbial growth of *P. stutzeri* 5190 and *A. infectoria* NIS4 on PCA and PDA media with carbon black and green ink, respectively.

Incubation was 72 h at 37 °C and 28 °C. Table 2 shows that tattoo tests with green and black inks had no or very little microbial growth. Tests with pure *P. stutzeri* 5190 and *A. infectoria* NIS4 cell cultures showed moderate and abundant microbial growth, respectively. This was confirmed by observations on Petri dishes (Figure 9).

### 2.4. Pool Enzymatic Profile on Activated Biogel

The results of the multiple enzymatic activities (19 enzymes by Api-Zym test) on the biogel and delivery residues were inconclusive both for bacteria *P. stutzeri* and fungal *A. infectoria* cell cultures, respectively. After seven days, the biogel-activated bacteria were extracted using a cotton swab. Subsequent analyses could not determine the presence or absence of the enzyme or the quantity of the enzyme expressed. In these instances, the residual traces of raw green ink impeded the accurate interpretation of the enzymatic reaction within the strip’s mini-vessel. The cause may be due to the presence of the color ink or complex and toxic substances (Cu ion) that can interfere with and inhibit enzymatic processes, as well as the regular reaction between the enzyme and substrate. Despite applying a 10-fold serial water dilution to the biogel removed from the pork rind, the results obtained on the seventh day were inconclusive. See Figure S4.

By contrast, the results of the separate tests determining lipolytic and proteolytic activity in the same biogel-activated bacteria and fungi were satisfactory and positive. These aspects are of great importance because they could be associated with the ability of pure microbial cells to interfere with the organic composition of pork rind surfaces, thereby facilitating the addition of raw inks.

### 2.5. Green Ink Removal on Pork Rind by Pure Microbial Cultures

Following these results, experimentation progressed by setting up in vitro tattoo ink bioremoval tests on pork rinds by adding *P. stutzeri* 5190 bacteria viable cells mixed with carbogel as a delivery system to the surface [65,66,67]. The same trials were carried out with a fungal culture of the *A. infectoria* NIS4 strain.

**Figure 10:**
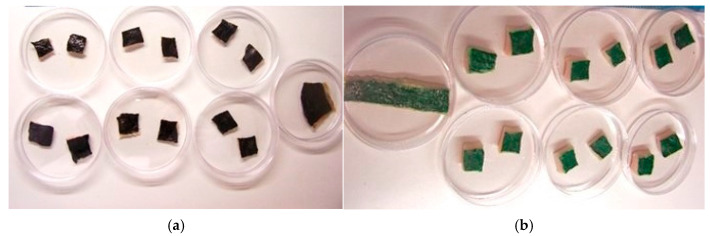
In vitro bioremoval ink tattoo on pork rind, with carbon black (**a**) and green (**b**) ink addition, in Petri dishes, in duplicate test.

Figure 10 shows carbon black and green inks in Petri dishes containing pork rind samples and pure culture microbial cells under closed environmental conditions.

**Figure 11:**
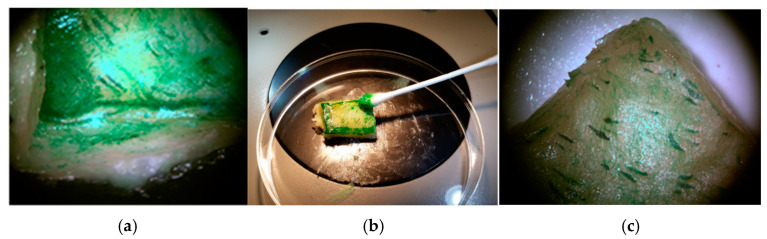
Viable cell bioactivity (activated biogel) of *P. stutzeri* DSMZ 5190 strain at 7th day on green tattooed pork rind (**a**); green ink tattoo removal by cotton swab (**b**); stereomicroscope surface observations after biogel removal (**c**).

The sequence of the biotreatment steps included applying *P stutzeri* DSMZ 5190 cells in activated biogel to the rind sample surfaces with the previously added green ink, as shown in Figure 11 and Table 3.

On the seventh day, the color changes in the lab-scale biogel *P. stutzeri* DSMZ 5190 strain biotreatments were measured; the results are reported in Table 3.

The partial color differences (∆L*, ∆a*, and ∆b*) and total color difference (∆E*ab) between the two samples were calculated.

Comparing color values before and after the carbon black and green tattooing treatments on the pork rind surface samples (Control 1, after versus before; AC vs. BC) showed no significant differences (*p* > 0.05). In fact, the lightness values changed only slightly from their original ∆E*ab values of 1.47 and 1.34. These responses are important because the trials were performed to test the stability of the chemical components, and spontaneous loss and smoothness phenomena can occur. In these cases, the pork rind surfaces lacked any surface alterations because of the absence of the eventual treatments (physical and chemical applications); thus, we did not expect any changes.

Conversely, on the seventh day, when the biotreatment (BT) with *P. stutzeri* DSMZ 5190 cells was performed to compare the green pork rind surfaces (A, after cleaning) with the same green ink surface at time zero (B, before cleaning), remarkable and statistical differences in lightness were recorded (*p* < 0.05). In fact, the partial color differences (∆L*, ∆a*, and ∆b*) and total color difference (∆E*ab) (BTA vs. BTB) were calculated with a ∆L* lightness value of 26.40 and a ∆E*ab value of 28.80. See Table 3.

Finally, in parallel tests, color measurements for the biotreatment with viable microbial cells were performed to compare the carbon black pork rind surfaces (BTA, after cleaning) with the same carbon black ink surface at time zero (BTB, before cleaning) on the seventh day. No remarkable differences in lightness were recorded (*p* > 0.05). In fact, the partial color differences (∆L*, ∆a*, ∆b*) and total color difference (∆E*ab) (BTA vs. BTB) were calculated with a ∆L* lightness value of −0.45 and a ∆E*ab value of 2.54.

Table 4 shows the results of the biotreatment trials carried out with *A. infectoria* NIS4 fungal cells in activated biogel, under the same operative conditions as above.

On the seventh day, the results from the fungal cultures of the *A. infectoria* NIS4 strain added to biogel on the pork rind surface were very similar to those described for the *P. stutzeri* 5190 DSMZ bacterial cells.

Under our experimental conditions, when included in carbogel as a delivery system, both pure viable *P. stutzeri* 5190 DSMZ cells and the *A. infectoria* NIS4 fungal strain exhibited positive and potential performance in removing green tattoo ink from the pork rind surfaces on the seventh day. These strains showed a loss of adhesion in the primitive tattoo inks used on the pork rind, facilitating soft mechanical ink removal using a cotton swab.

*P. stutzeri* 5190 strain (from DSMZ microbial collection) is classified as safe bacteria and included in Risk group 1 (+) (classification according to German Technical Rules for Biological Agents, TRBA); furthermore, there are no known Nagoya Protocol restrictions for this strain. Moreover, the fungi *A. infectoria*, NIS4 strain, is included in Risk group 1 (+) (classification according to German TRBA); however, some strains are potentially able to induce pathogenic forms like chronic rhinitis in humans.

The preliminary chemical characterization of the ink compositions using Py-GC/MS, NMR, and FTIR suggest potential changes in the pigments in the green ink and organic compounds adopted as binders (glycerin and additives), even in trace amounts. These results were more evident when the tattoo inks were submitted to enrichment culture techniques in environmental microbial samples, that is, the soil and composted residues. To determine how the green ink applied as a tattoo on the pork rind surface was removed, it is necessary to consider at least three hypotheses: (i) the natural composition of the pork rind; (ii) the application mode and chemical composition of the inks; and (iii) the performance of the viable microorganisms (bacteria and fungi).

Given the considerable number and complexity of the phenomena associated with these hypotheses, we believe that further in-depth investigations are appropriate to shed light on certain aspects of the applied research presented here.

The experiments conducted here must be considered entirely preliminary. The results obtained with the use of pork rinds purchased from large-scale retail outlets provide a useful model for initial trials on a laboratory scale. However, these results cannot yet be applied directly to human skin, as safety requirements must first be met, and bioethical standards associated with any experimentation on humans and/or living animals must be correctly applied.

### 2.6. Electrochemical Removal of Green Ink from Wastewater

Following the experiment (Section 3.1), the research activities carried out at the lab scale using ink tattoos produced several wastewater effluents and solid residues, which were stored and quantified. The main chemical properties of the wastewater effluents (for example, containing green ink residues) showed the following values: BOD_20_ = 70.3 mg O_2_ L^−1^ and COD = 12,700 mg O_2_ L^−1^. As a result, the biodegradability index (BOD_20_/COD) was <0.1, indicating an effluent rich in chemical pollutants and xenobiotic and recalcitrant substances.

An electrochemical treatment was adopted. Tests carried out on the green tattoo ink wastewater solution demonstrated the electric current density’s ability to pass through the liquid, closing the electric circuit/ring between anode and cathode (represented in this study by a graphite electrode and an iron coin, respectively). Near the cathode surface, many microbubbles were produced because of electron flow on the cathode surface and the co-translocation of Cu ions—the core of the green ink complex—from the liquid phase to the coin surface, taking on the same green color.

**Figure 12:**
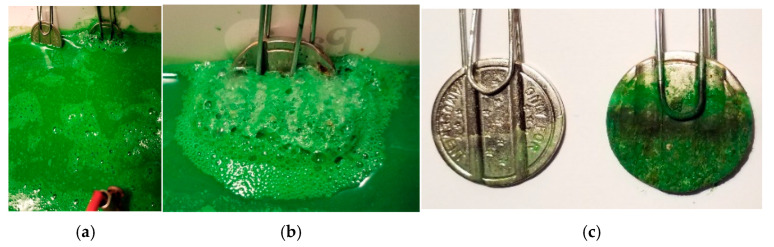
Electrochemical trials carried out on green ink tattoo in wastewater solution; (**a**) reference coin without current; (**b**) coin electrode under power working phase; (**c**) comparison view between two coins at end of trial.

The electric current did not alter the high stability of the green ink’s molecular structure, as confirmed by our chemical analyses. Reactions at the only Cu ion in the organic–metal complex within the green ink did not change its structure. The green organo-metallic complex migrated from the circulating solution to the surface of the metal coin, which changed color and acquired a homogeneous green coating. The green coating deposition on the metal coin was quantified after 90 min using an electrochemical working cell; the coin increased its total dry weight to 1.33 g after drying in an oven at 40 °C for 24 h. Figure 12 shows the results of the electrochemical treatment of the green ink wastewater solution at the laboratory scale.

Wastewater treatment plants process polluted effluents, including inks, using a combination of physical and chemical (acid and basic reagents, among others, to induce neutralization, precipitation, flocculation, and sanitization) and biological (under aerobic conditions like activated sludges, or anaerobic facultative nitri-denitrification processes, and under anaerobic conditions like anaerobic reactors) treatments. These processes are usually applied in sequence, though they can also be used individually. It is generally acknowledged that these processes result in significant reductions in the levels of the original pollutants. However, they have not been shown to be effective in the treatment of xenobiotic compounds (such as inks) even at low concentrations and as cocktail mixtures. For these reasons, an electrochemical process could be an effective solution for selectively reducing pollution and stable compounds in effluents, while also allowing for the fast recovery of the main compounds.

Using low electric current considerably reduced the amount of green ink residues in the wastewater at the lab scale. This result will permit us to plan a new and better intervention to minimize the negative environmental impact caused by our research.

### 2.7. Environmental Impact, CO_2_ Equivalent Emissions, and Mitigation Actions

Based on previous studies, we will now evaluate the environmental impact of this study, its CO_2_ equivalent emissions, and mitigation actions undertaken by planting new plants [68].

The environmental impact of this study in terms of social cost and CO_2_ eq. emissions was EUR 168.0 and about 1200 kg CO_2_ eq., respectively. As mitigation action, we assumed that 100 kg of CO_2_ eq. emissions would be converted into biomass via natural photosynthetic processes over 10 years, with one new plant planted in soil [68]. This led us to plant new *Quercus* plants for mitigation purposes. Specifically, we used *Q. robur* L. and *Q. cerris* L., both mycorrhized by *Tuber aestivum*; these plants are known for their ability and potential to produce truffles in soil symbiosis roots. They are autochthonous species with a significant ability to adapt to their environment. A QR code tag linked to this manuscript was added to twelve new plants at Green Park—Campus Unimol (41°61′40″ N 14°30′80″ E).

## 3. Materials and Methods

### 3.1. Experimental Design

**Figure 13:**
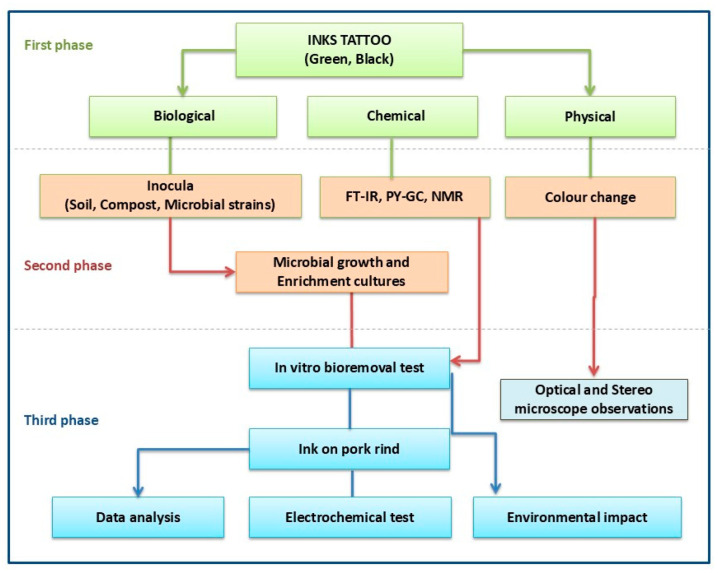
Schematic diagram of experimental design.

The experimental design of the entire trial is shown schematically in Figure 13.

The first two phases were dedicated to comprehensively examining the chemical, physical, and biological aspects of the process to identify the specific chemical components and develop a biologically enriched culture to effectively clean the inks used. The third and final phase, as illustrated in Figure 13, was dedicated to an in-depth analysis of the environmental impact, the results obtained, and a detailed discussion of the electrochemical and biological removal treatments.

### 3.2. Tattoo Inks

The following two tattoo inks were selected (Figure S5), and the chemical composition of their technical sheets are reported.

“Azayaka True Green dispersion”: Pigment Green 7 (CI 74260, Phthalocyanine Green), Pigment Yellow 1 (CI 11680, 2,2′-[(3,3′-dichlorobiphenyl-4,4′-diyl)didiazene-2,1-diyl]bis(3-oxo-N-phenylbutanamide); water, glycerine, isopropyl alcohol, food-grade rubber, phospholipids, and food-grade emulsifier (I Max, Riccione, Italy).

“Makkuro Sumi Pure Black tattoo ink”: Water, glycerine, isopropyl alcohol, phospholipids, food-grade emulsifier, and carbon black (CI 77266) (I Max, Riccione, Italy).

Furthermore, Kremer “23000 Phthalo Green Dark” and Zecchi “Primary Hansa Yellow” commercial products were adopted and analyzed as references (I Max, Riccione, Italy).

### 3.3. Chemical and Physical Parameters

The following chemical and physical parameters were determined according to standard methods [69,70]: pH, total solids (TS), volatile solids (VS), chemical oxygen demand (COD), biological oxygen demand (BOD_5_ and BOD_20_), and biodegradability index (BOD_20_/COD).

### 3.4. Chemical Analyses Using Infrared Spectroscopy

Fine powders were obtained by drying aliquots of the two tattoo inks (green and black) at 28 °C for ten days. Then, ink samples were analyzed using Fourier transform infrared (FTIR) spectroscopy in ATR (attenuated total reflectance) mode to determine their composition [50]. Spectra were recorded using a Thermo Scientific Nicolet iS10 instrument (Waltham, MA, USA) equipped with a diamond crystal and a DTGS detector in a range of 4000–600 cm^−1^, with a resolution of 4 cm^−1^ and 32 scans; a background analysis was carried out periodically, and the spectra obtained were used to correct those of the samples. The spectra were interpreted by comparison with paper databases and the literature.

### 3.5. Chemical Characterization by Py-GC/MS

A Pyroliser PY3030 from Frontier Laboratories Ltd. (Fukushima, Japan), coupled in-line with a Gas Chromatograph 8890N and a mass spectrometer detector 5977B (Py-GC/MS by Agilent Technologies, Palo Alto, CA, USA), was used for pyrolysis to analyze the organic fractions of the ink samples for the fundamental identification of the components and substances present. Micrograms of each ink sample were mixed with 2.0 µL of hexamethyldisilazane (HMDS) and placed in a quartz tube. The detailed working conditions have been published elsewhere [71]. Py-GC/MS analyses were performed to identify the composition of the ink samples.

Instrumental operating parameters: Analytical pyrolyzer EGA-Py-3030D (Frontier Laboratories Ltd.); pyrolysis furnace temperature: 550 °C, 0.2 min; interface temperature: 280 °C; Gas-Chromatograph 6890 (Agilent Technologies); injection: split mod., 280 °C, ratio 5:1; carrier gas: He (1 mL min^−1^); column: HP-5MS (30 m × 0.25 mm, film thickness 0.25 μm, Agilent Technologies); oven temperature: 36 °C for 10 min and 10 °C min^−1^ until 280 °C; then, 2 min, and 20 °C min^−1^ until 300 °C for 20 min. Transfer line: 280 °C.

Mass spectrometer 5973 (Agilent Technologies); ionization: EI negative mod. 70 eV; range *m*/*z*: 50–600; ion source: 230 °C; quadrupole: 150 °C.

The samples were pyrolyzed in the presence of a derivatizing agent (hexamethyldisilane, HMDS).

### 3.6. Sample Preparation and NMR Experiments

For NMR experiments, a 20 µL ink sample (green ink) was solubilized in D_2_O solution [containing 1 mM of sodium 3-trimethylsilyl [2,2,3,3-^2^H_4_] propionate (TSP) for ^1^H spectral reference], obtaining a volume of 700 μL. Ink samples previously enriched by soil and compost inocula were collected in duplicate and analyzed. Samples were transferred into NMR tubes.

One-dimensional ^1^H NMR (1D) spectra were recorded on a Bruker Avance III-600 MHz spectrometer (Bruker BioSpin GmbH, Rheinstetten, Germany) at 27 °C using a TCI CryoProbe with a Z gradient. The excitation sculpting pulse sequence moderated the water signal in the 1D spectra. Acquisition parameters and data manipulation were the same as those reported in Saviano et al. [72]. For resonance assignments, two-dimensional (2D) clean total correlation spectroscopy (TOCSY) and 2D natural abundance were applied.

^1^H–^13^C heteronuclear single quantum coherence (HSQC) spectra were acquired. Acquisition parameters and data manipulation were as previously reported [72]. ^1^H spectra in water were referred to an internal 0.1 mM TSP and assumed to resonate at 0.00 ppm; ^13^C spectra in water were referred to an internal 0.1 mM CD_3_OD and assumed to resonate at 49.00 ppm.

### 3.7. Tattoo Device

A tattoo machine (typically called a “gun”) is a manual electric device used to create body decorations using a system of electromagnetic coils that determine the linear movement of a metal bar inside, placing one or more needles that penetrate the skin to deposit pigment in the dermis. The speed at which the needles penetrate the epidermis is about 50 penetrations per second. A modern device employs a “rotary” electric motor with variable power, driven by a foot pedal or wirelessly, and a central body containing the needles. The depth reached by the needles is approximately 2–3 mm.

**Figure 14:**
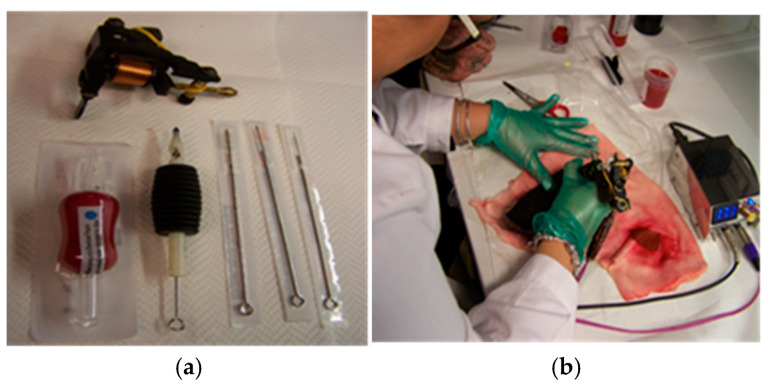
Tattoo machine and needles used in this work (**a**); tattooing pork rind (**b**).

In our trials, two types of needles were used: a 13R model (Round Liner, 0.40 mm) for tracing edges and a 15 M model (Magnum, larger flat needles) for filling, supported by an electric device working at 22 μF power capacitor (Spina model, Itattoo, Napoli, Italy). The pork rind tattooing process was carried out by a person with substantial experience in the field, having worked in it for over ten years; see Figure 14.

### 3.8. In Vitro Tattoos on Pork Rind, at Lab Scale

The bioremoval protocol included manually tattooing the pork rind surface to simulate a human’s tattoos. Pork rind samples were purchased by GDO from food channels (Conad, Pescara, Italy) and stored at 4 °C for three days before use.

At the lab scale, large tattoos were applied (carbon black ink: 19.0 × 7.5 cm and 13.5 × 11.0 cm; green ink: 13.5 × 11.0 cm and 10.5 × 7.0 cm). Then, the tattooed pork rind surface was cut to obtain smaller samples (about 2.0 × 2.0 cm) appropriate for each treatment, in duplicate.

Prior to the tattooing process, all pork rind samples were placed in a thermostatic room at 20 °C for 30 min. The tattoo inks were diluted in a 50:50 *v/v* ratio with sterile water at 20 °C immediately before commencing the tattoo treatments. The average time required to ink a tattoo on a pork rind was approximately 30 min for a 100 cm^2^ area. The temperature of the pork rind was monitored at regular intervals and found to be within two degrees of the laboratory room temperature.

Before the tattoo ink was applied, great care was taken to ensure that the pork rind was washed with neutral antimicrobial soap (0.55% Benzalkonium chloride) followed by a further cleaning treatment with cotton and 70% isopropyl alcohol (5 min of contact). The same chemical cleaning protocol was adopted for the pork rind after the tattoo application.

### 3.9. Microorganisms, Cultural Media, and Environmental Inocula

Table 5 illustrates the microbial inocula employed in the in vitro bioremoval test at the laboratory scale. Two microbial strains already known and adopted in previous studies were considered: a bacterial strain, *P. stutzeri* DSMZ 5190 [58], from the DSMZ collection (https://www.dsmz.de/collection, accessed on 17 July 2024) and a fungal strain, *A. infectoria* NIS4 [60].

Furthermore, two environmental samples were used as microbial inocula for enrichment culture techniques: a soil mix (S) and a composted extract sample (C), previously characterized, adopted, and described by the present authors [73]. Two grams of each environmental sample was added separately to a water suspension containing the two inks (1.0 mL of each ink in 50 mL of sterile M9 mineral salt medium [74], in 100 mL useful-volume PET vials). Control tests without soil and composted residue inocula were carried out. Tests were performed under constant temperature conditions in a laboratory incubator at 20 °C. At regular intervals (3 months), aliquots of cultures from previous broths (10 mL) were transferred to PET vials containing new fresh sterile M9 medium. Under these predefined conditions, the enrichment cultures were performed for 12 months.

For evidence and to numerate the total viable aerobic microbial counts, both M9 mineral salt agarized medium and Plate Count Agar (PCA) (Oxoid, Hampshire, UK) were adopted with an incubation temperature of 37 °C for 72 h. Peptone Dextrose Agar (PDA) and WL (BD Difco™, Milano, Italy) media were preferred, both for their mycete counts (fungi and yeasts) and for the associated microbial isolation technique; incubation was performed at 28 °C for 48 h.

Bacterial and fungal counts were expressed as Colony-Forming Units (CFUs) per 1.0 mL broth culture, and the putative bio-degradative ability of the microbial isolates was determined. Physical (color, supernatant, sediment, and turbidity) and chemical changes and biological profiles were recorded [74].

Carbogel (CTS, Albavilla Vicentina, Italy) was adopted as a delivery system both with *P. stutzeri* and *A. infectoria* viable bacteria and fungi cells, respectively [65,66,67]. Before use in the treatment, the cells were grown in media. After centrifugation and before mixing with the carbogel powder (2.0% *w*/*w*), a cell pellet was suspended in a physiologically sterile solution (NaCl 0.9 g L^−1^) to obtain a biogel with 10^7^–10^8^ UFC g^−1^ of viable cells. Treatments with the carbogel but without microorganisms were performed as a control test.

Multiple enzymatic activities of the pure microbial cultures were determined using the API-ZYM system (Bio-Merieux Italia, Bagno a Ripoli, Italy). A semi-quantitative evaluation of the activities of 19 hydrolytic enzymes was conducted according to Viti et al. [75] and Marasco et al. [76]. All determinations were made with three replicates. The reproducibility was >95%.

In addition, lipolytic and proteolytic activities were determined according to a previous study [60].

### 3.10. Extraction of Genomic DNA from Inocula Samples

We then assessed the bacterial diversity and taxonomic composition through Next-Generation Sequencing (NGS). DNA was extracted at the end of the experiment on the enriched ink solutions inoculated by the soil and compost samples subjected to the different treatments using the E.Z.N.A.^®^ Soil DNA Kit (Omega Biotek, Norcross, GA, USA), following the manufacturer’s instructions. The fungal communities’ DNA was extracted for analysis using the DNeasy 96 PowerSoil Pro QIAcube HT Kit (QIAGEN, Hilden, Germany), according to the manufacturer’s instructions.

### 3.11. Optical Microscope Observation

We adopted the laboratory model of the optical OM-Nikon Eclipse E600 model (Nikon Instruments Europe B.V., Amsterdam, The Netherlands) and the stereo SM-Zeiss AxioScope (Carl Zeiss Spa, Milan, Italy) microscope, connected to high-resolution digital cameras. Fragments of pork rind surface samples before and after set tattooing intervals were biocleaned and then examined.

### 3.12. Color Measurements

**Figure 15:**
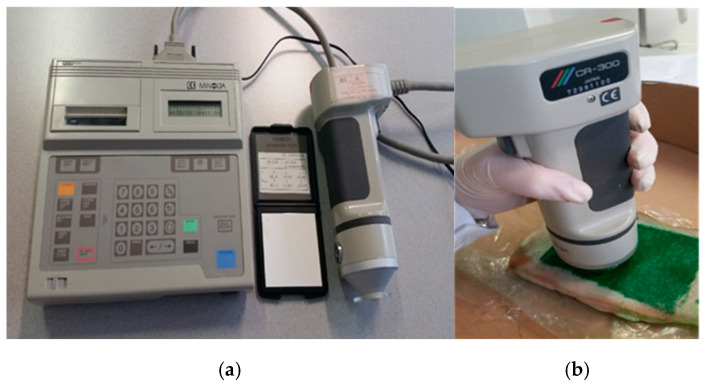
Colorimeter adopted in all the trials (**a**), working on tattooed pork rind (**b**).

The Miniscan Konica CR 300 Minolta model (Minolta, Milano, Italy) was adopted to evaluate the efficiency of the biotreatment; see Figure 15. The chromatic coordinates of the pork rind surfaces were determined: L* (lightness axis moves from a top value of 100 = white to a bottom value of 0 = black), a* (axis is associated with changes between red and green), and b* (axis is associated with changes between yellow and blue). According to the CIELAB 1976 system, the partial color differences (∆L*, ∆a*, and ∆b*) and the total color difference between the two samples (∆E*_ab_ [(L*)^2^ (a*)^2^ (b*)^2^]^1/2^) were calculated [77,78].

Color measurements were made on the surfaces of the tattooed pork rind samples before (TBB) and after (TAB) biocleaning. In addition, color measurements were calculated for untattooed cut pork rind surfaces (after, as control; AC) vs. untattooed pork rind surfaces (before, as control; BC). Data concerning color measurements and biochemical responses were submitted for statistical analyses (Student’s *t*-test) using the SAS statistical software (SAS/STAT v15.1 2018).

### 3.13. Electrolytic Equipment Adopted for Green Ink in Water Solution

The experiments used an operating electrolytic unit (De Ponti Electronics, Treviglio, Italy) to evaluate an electrochemical treatment for removing green ink from an aqueous solution. The preliminary system used for our tests included a single chamber filled with 200 mL of tap water and 20 mL of green tattoo ink. The chamber (PET; 20 × 15 cm; total useful volume of 500 mL) included a pair of electrodes working via the regulation at several electric power values. The final pH solution was corrected to 4.5 by acetic acid to increase conductivity to 800 µS/cm (GroLine HI991301 and HI98331 models, Hanna Instruments, Padova, Italy). First electrode (anode): graphite cylindrical shape, 10 cm length, 3 mm diameter. Second electrode (cathode): an iron coin, 2.0 cm diameter, 5.45 g of weight. Distance between electrodes: 20 cm. Intensity of current applied: 200 mAmpere, voltage 12 Volts, 50–60 Hz. Treatment time: 90 min. The temperature of the treated ink mixture was measured over time using a thermometer (GroLine HI9814 model, Hanna Instruments, Padova, Italy).

### 3.14. Environmental Impact

To reduce environmental impacts originating from these experiments, a preliminary assessment of the inputs (energy consumption, reagents, and materials at the lab level) and outputs was first converted into CO_2_ equivalents (CO_2_ eq.) and then into partial mitigation action by planting plants, according to our previous work [67]. The plants were furnished by Vivaio Forestale Regionale “Selva del Campo”, Campochiaro, Italy.

## 4. Conclusions

To develop an innovative biological procedure to remove ink from the human body, it is essential to conduct comprehensive investigations into the biochemical aspects and complexity of the associated phenomena.

We extensively characterized green tattoo ink (Hexadecachlorinate copper phthalocyanine—C_32_Cl_16_CuN_8_) through Py-GC/MS, NMR, and FTIR analyses. The results demonstrated intriguing profiles related to the primary chemical components. Two microbial strains, namely, *P. stutzeri* 5190 DSMZ viable cells and the *A. infectoria* NIS4 strain that had previously been isolated and identified were employed as biocleaning agents on tattooed pork rinds, used to simulate human tattoos. Under our experimental conditions, both microorganisms demonstrated the potential to remove green tattoo ink. Finally, the results of an electrochemical treatment on green ink wastewater offered a potential solution for cleaning chemical residues.

To conclude, these results offer a promising starting point for further investigations into the potential of innovative biological methods for removing tattoo inks from human skin and wastewater. We will subsequently investigate the efficacy of different methods for deep tattoo removal using the same pork rind matrix and relate the current results to the existing literature.

## Figures and Tables

**Table 2:** Microbial growth of pure cultures and enriched environmental soil and compost inocula on M9 medium, rich media (Plate Count Agar (PCA) and PDA), with and without additions of ink tattoo (treatment and control, respectively), after 72 h, in duplicate Petri dishes.

	*P. stutzeri* 5190DSMZ		*A. infectoria* NIS4		Soil, Compost Inocula	Ink Tattoo
Added Ink	Yes	No	Yes	No	Yes	Green
M9	+	−	+	−	+/−	
PCA	+	++	nd	nd	−	
PDA	nd	nd	++	++	−	
M9	+	-	+	-	+/−	Carbon black
PCA	+	++	nd	nd	−	
PDA	nd	nd	++	++	−	

Legend: − absent; −/+ very low; + middle; ++ abundant; nd: not determined.

**Table 3:** In vitro color measurements on pork rind surface tattooed samples added with *P. stutzeri* DSMZ 5190 strain in “Biogel Treatment After” (BTA) vs. “Biogel Treatment Before” (BTB), and control (C), “After” (AC) vs. “Before” (BC). The values at 7th day obtained as mean, 2 replicates, and 12 color measures for each. Significance was evaluated by Student’s *t*-test.

Ink	Color Change	BTA vs. BTB	*p*	AC vs. BC	*p*
Green	ΔL*	26.40	0.032	−1.13	0.045
	Δa*	8.90	0.040	0.32	0.038
	Δb*	5.40	0.048	0.90	0.032
	∆E*_ab_	28.80	0.026	1.47	0.028
Carbon black	ΔL*	−0.45	0.078	−0.75	0.065
	Δa*	−1.93	0.082	−0.36	0.077
	Δb*	−1.57	0.065	1.05	0.091
	∆E*_ab_	2.54	0.088	1.34	0.090

**Table 4:** In vitro color measurements on pork rind surface tattooed samples added with *A. infectoria* NIS4 strain in “Biogel Treatment After” (BTA) vs. “Biogel Treatment Before” (BTB), and control (C), “After” (AC) vs. “Before” (BC). The values at 7th day obtained as mean, 2 replicates, and 12 color measures for each. Significance was evaluated by Student’s *t*-test.

Ink	Color Change	BTA vs. BTB	*p*	AC vs. BC	*p*
Green	ΔL*	25.30	0.037	−1.40	0.038
	Δa*	7.80	0.048	0.28	0.042
	Δb*	4.90	0.038	0.65	0.049
	∆E*_ab_	29.20	0.026	1.33	0.023
Carbon black	ΔL*	−0.40	0.088	−0.65	0.068
	Δa*	−1.65	0.091	−0.42	0.084
	Δb*	−1.33	0.075	1.00	0.062
	∆E*_ab_	1.90	0.082	1.18	0.080

**Table 5:** The list of inocula adopted for the experimental trials with ink tattoo.

Codes	Treatments	Details
0	Control	No ink addition
S	Soil mix	Soil sample extract [73]
C	Compost mix	Commercial compost extract [73]
Ps	Bacterial strain	Pure culture *P. stutzeri* 5190 strain DSMZ [58]
Ai	Fungal strain	Pure culture *A. infectoria* NIS4 strain, Dpt. Science, Unimol, Italy [60]

## Data Availability

Data are contained within the article and Supplementary Materials.

## Supplementary Materials

The following supporting information can be downloaded at https://www.mdpi.com/article/10.3390/molecules29235543/s1, Figure S1: Two-dimensional TOCSY superposition; Figure S2: HSQC experiment; Figure S3: Absence of bacterial and fungal growth on PCA and WL media; Figure S4: Api Zym test; Figure S5: Tattoo inks adopted in this work; Table S1: List of peaks in the green ink sample and attribution to PG7 and PY1 references.
