# Supplementary material for: Investigation on Tattoo Ink (Hexadecachlorinate Copper Phthalocyanine) Removal: Novel Chemical and Biological Approach
Source: Molecules. 2024 Nov 24;29(23):5543. doi: 10.3390/molecules29235543 (PMC11644021; doi:10.3390/molecules29235543)
Supplement: Supplementary file 1 [file molecules-29-05543-s001.zip › molecules-3261571-supplementary.pdf]

molecules

MDPI

Article

Investigation on Tattoo Ink (Hexadecachlorinate Copper Phthalocyanine) Removal: Novel Chemical and Biological Approach

Giancarlo Ranalli[1], Alessia Andreotti[2], Maria Perla Colombini[2], Cristina Corti[3], Laura Rampazzi[3,4], Gabriella Saviano[1,5], Debora Paris[5] and Claudio Caprari[1,*]

SUPPLEMENTARY MATERIALS

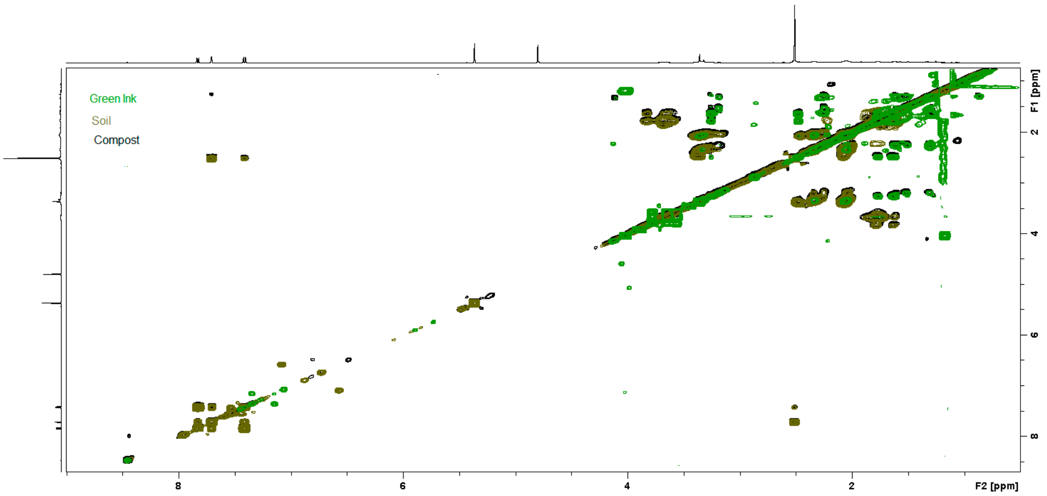

Figure S1. 2D TOCSY superposition of all three experiments: raw green ink (green), soil after treatment with green ink (brown), and compost after treatment with green ink (black).

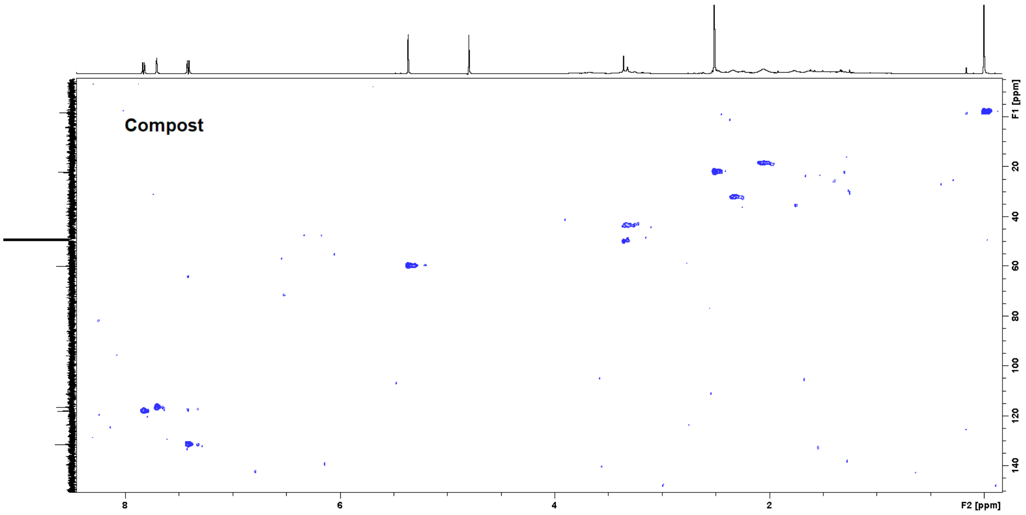

Figure S2. HSQC experiment of compost after treatment with green ink.

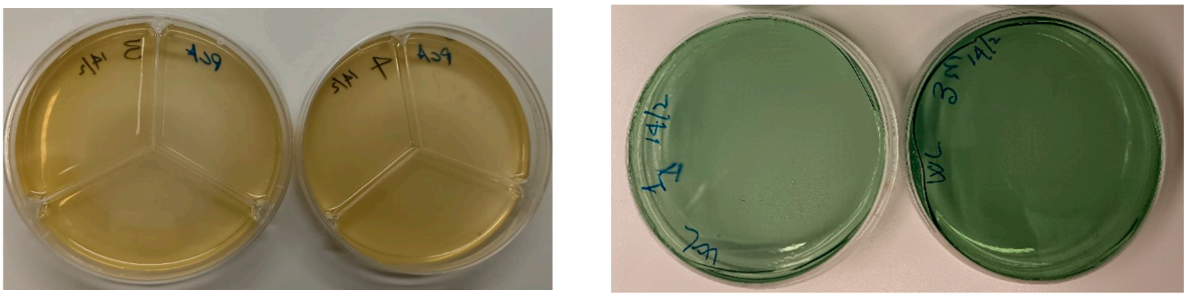

(**a**) (**b**)

Figure S3. Absence of bacterial and fungal growth on PCA and WL media, after 72 hours of incubation, at 37 °C and 28 °C., respectively, by inoculum of enrichment cultures.

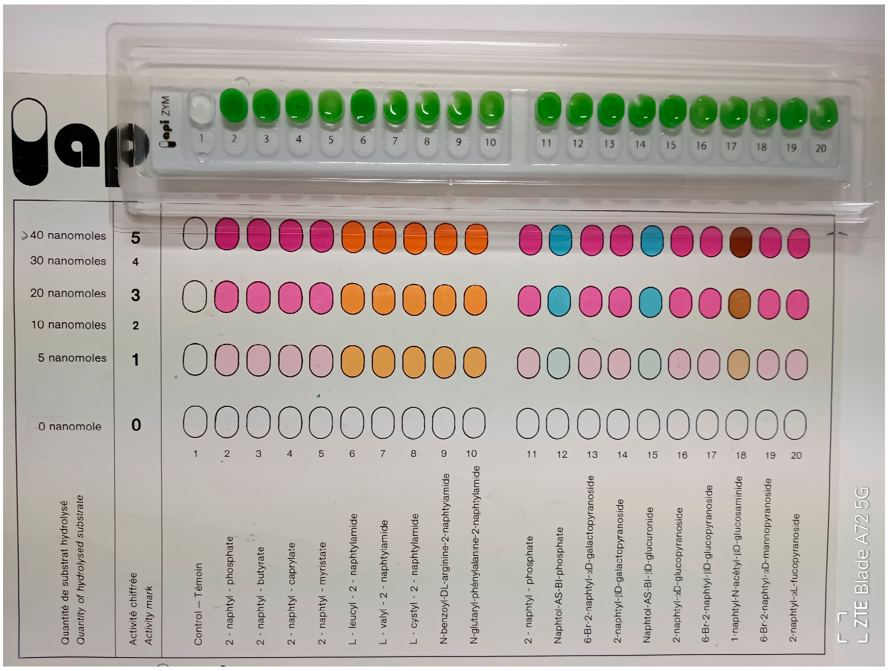

Figure S4. Api Zym test at 7th day, on the biogel-activated bacteria.

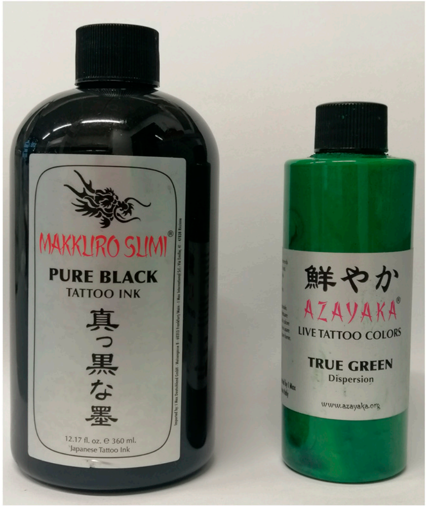

Figure S5. Tattoo inks adopted in this work.

Table S1. List of peaks in the green ink sample and attribution to PG7 and PY1 references.

Green ink - Azayaka True Green dispersion (cm^{-1})	PG7 - Kremer 23000 Phthalo Green dark (cm^{-1})	PY1 - Zecchi Primary Hansa Yellow (cm^{-1})
3243		3241
3184		3182
3144		3142
3106		3102
3011		3011
2879		2872
2812	2814	
2692	2689	
2634	2632	
2575	2576	
2486	2488	
2432	2434	
2389	2389	
1665		1666
1618		1618
1600		1600
1533		1532
1505		1505
1491		1491
1464	1463	
1449		1449
1404		1405
1342		1342
1306	1304	
1291		1292
1236		1236
1211	1208	
1175		1174
1152	1151	
1138		1136
1112	1114	
1095	1094	
1062		1064
1023		1023
998		999
969	967	
924		924
914		914
871	873	
848		848
757		757
747	747	
698		698
625		624

Reports the peaks that can be attributed to one of the reference pigments, either PG7 (Kremer 23000 Phthalo Green dark) or PY1 (Zecchi Primary Hansa Yellow). Peaks common to both reference pigments and those not clearly attributable to either are omitted.
